# Lactation induction, shared breastfeeding, and the right to opacity: a reflection on the use of health protocols and practices for women who have relationships with women

**DOI:** 10.31744/einstein_journal/2026RW2297

**Published:** 2026-08-03

**Authors:** Fernanda Evangelista Bandeira de Melo, Fernanda Baeza Scagliusi

**Affiliations:** 1 Faculdade de Saúde Pública Universidade de São Paulo São Paulo SP Brazil Faculdade de Saúde Pública, Universidade de São Paulo, São Paulo, SP, Brazil.

**Keywords:** Lactation, Clinical protocols, Breast feeding, Sexual and gender minorities, Homosexuality

## Abstract

Lactation induction is the process of stimulating milk production when pregnancy has not occurred, but there is still a desire to breastfeed. Initially designed for adoptive mothers and mothers through surrogacy, the protocol does not explore other uses, such as in cases of LGBTQIA+ families, in which breastfeeding may also be a significant symbol in the transition to parenthood. Thus, we seek to explore the complexities of the use of lactation protocols by cisgender women couples who have chosen co-lactate. This article is based on qualitative research conducted through semi-structured interviews with women in São Paulo and Rio de Janeiro, Brazil, between June and October 2024. Seven women participated: three couples and one non-gestational mother. From this analysis, two analytical categories emerged: the first addresses the sources of information used by the participants, highlighting the key role of information networks built through the internet and the notable absence of healthcare professionals as references in this process. The second category discusses the participants’ experiences with the induction protocol, highlighting the challenges of balancing their routines with the protocol’s demands and the importance of healthcare professionals who act as positive deviants from the hegemonic model of care. We propose a reflection within the field of healthcare on its onto-epistemological assumptions, suggesting the right to opacity as a guiding principle in the construction of healthcare practices, acknowledging that it is not necessary to understand the other – or their life logics – in order to form a care alliance that goes beyond rigid, standardized protocols.

## INTRODUCTION

Lactation induction is the procedure used to stimulate milk production in situations where pregnancy has not occurred, yet there is still a desire to breastfeed.^(1)^ The most commonly used protocol originates from the personal experience of lactation educator Lenore Goldfarb, in partnership with pediatrician Jack Newman, who guided her through the process. Published in the document *“*Newman-Goldfarb Protocols for Induced Lactation®: The Guide for Maximizing Milk Supply*”,*^(2)^ the text provides general information about breastfeeding and outlines induction recommendations through three main protocols: the regular protocol, the accelerated protocol, and the protocol for individuals experiencing menopause.

Initially designed for adoptive mothers and those who become mothers through surrogacy, the protocol provides guidelines that combine hormone therapy with mechanical stimulation using a breast pump. Although these guidelines serve as a starting point for individualized induction processes, the protocol does not mention or consider other possible applications – such as in cases of LGBTQIA+ parenthood. In response to this gap, more recently, the Academy of Breastfeeding Medicine^(1)^ developed a protocol aimed at addressing breastfeeding care for sex-gender dissident groups. While it does not propose a new induction protocol per se, it draws attention to central aspects of healthcare for LGBTQIA+ populations, such as the appropriate use of pronouns, specific considerations regarding hormone treatments, and care strategies for gender-diverse couples wishing to breastfeed.

It is important to note that the desire to breastfeed can arise from multiple sources, as breastfeeding carries different meanings shaped by personal histories and the ways in which individuals organize care within their communities. On the one hand, throughout modern history, breastfeeding has been predominantly constructed around the benefits it provides to the newborn^(3)^ – being understood as a source of nutrition, a protective factor against illness, and a promoter of overall health. However, breastfeeding may also hold different meanings and have different effects for those who choose to do it.

These meanings are also shaped by various representations constructed through health communication, social media, and lived experiences in one’s social territory, which collectively shape the symbolic and social value of breastfeeding as exemplified by the paradigm shift that repositioned it from a practice intended exclusively for subordinated women to one of caring for children’s nutrition, grounded in hygienist perspectives.^(3)^ Thus, these meanings often go beyond the biomedical perspective that frames breastfeeding primarily as a nutritional function and acquire symbolic significance that contributes to the desire – or lack thereof – to breastfeed. This broader understanding is essential in recognizing that, for LGBTQIA+ individuals, breastfeeding involves dimensions that exceed the physiological, acting as a meaningful symbol in the transition to parenthood.

Here, without claiming to provide a genealogical account, we highlight a few key historical markers: breastfeeding has historically been constructed as an exclusively female task, but also as a subordinated one, as it is associated with nature through the exposure of the body and, therefore, animalizes the body.^(4)^ As such, it was often assigned to poor women in Europe – as seen in 13th-century France with the so-called “wet nurses” – and to enslaved Black women in Brazil from the 18th century onward, shaped by colonial and enslavement processes.^(5)^ With the cholera and yellow fever epidemics of the 19th century, a new discourse emerged, influenced by the hygienist perspective of medical authority, which began to construct a new maternal ideal: the biological mother should be the primary caregiver, including being responsible for breastfeeding.^(5,6)^ It was also during this period that medical discourse began to emphasize the maternal and infant health benefits of breastfeeding, aiming to promote the practice. However, this discourse focused primarily – leaving lasting traces even today – on individual responsibility for the outcomes of breastfeeding or weaning, with little regard for the sociocultural factors that may facilitate or hinder sustained lactation.^(3)^

From the convergence of medical hygienist discourse and capitalist interests, infant formula emerged as a convenient solution for child feeding. Marketed as nutritionally equivalent to human milk, infant formula was presented as the ideal food for the modern woman. Its popularization – through advertising and mass media – was not accompanied by a critical understanding of the country’s social realities and the diverse needs of its population. Furthermore, the introduction of formula feeding has had a direct impact on early weaning.^(7)^

Although shared breastfeeding among female couples is a relatively recent practice, the act of breastfeeding a child one did not gestate is not new in Brazil. In addition to the “*amas de leite”* – Black women who breastfed white children within an exploitative relationship structured by slavery – the practice was also carried out by women in peripheral regions, framed within a logic of caregiving articulated through community networks. These networks give rise to alternative forms of territorial organization that are deeply intertwined with daily routines and lived experiences.^(5)^

In addition, the infant formula industry’s impact, public health policies, and consequently, health communication, have significantly influenced how different breastfeeding arrangements are perceived in Brazil. We highlight two key developments in this regard: first, the publication of Ordinance No. 1016 of 1993, which, through the implementation of the “Rooming-In” system, prohibited cross-nursing; and second, Ordinance No. 2415 of 1996, which further restricted cross-nursing based on recommendations from the National AIDS Commission.^(8,9)^ A more recent moment of change came with the subtle recognition by the State of breastfeeding as a tool for bonding – officially acknowledged in the Dietary Guidelines for Brazilian Children Under 2 Years of Age.^(10)^

We draw attention to these specific regulations to shed light on a new layer of the breastfeeding paradigm. On the one hand, human milk is positioned in health policies as a complete food – capable of protecting both the child and the breastfeeding person from illness and fostering bonds between them. On the other hand, there are structural aspects of life under capitalism that hinder the full exercise of breastfeeding. However, this alone is insufficient to fully understand how breastfeeding intersects with the lives and parenthood experiences of LGBTQIA+ individuals. Also relevant is the role of care and affective networks that are built through the act of breastfeeding – networks that are historically rooted in modes of producing care within territories where micropolitics are what sustain life.

### Some theoretical reflections

Thinking and producing research from the Global South, and directed at the Global South, requires acknowledging the impacts of the colonial wound on the ways of thinking and organizing life in the territory. To that end, we draw on authors who think through the lens of coloniality to reflect on the potentialities and limitations of the use of lactation induction protocols based on the experiences couples of cisgender women who chose to share breastfeeding, highlighting the tension between the possibilities arising from the use of medical technologies by LGBTQIA+ people and what Donnangelo calls social medicalization^*^.^(11)^ This is because while it is true that the modes of social and cosmological organization of modernity were violently imposed on colonized territories, it is also true that these territories carry, in their materiality, the memory of other ways of organizing life that, when reproduced, constitute a form of resistance to coloniality.^(12)^

The central distinction of coloniality, constituted through the imposition of homogeneous and separable categories, lays the foundation for the myth of a natural separation between humans and the environment. This distinction is also responsible for the dehumanization of all those who do not resemble the colonial subject, enabling their reduction to something less than human, justifying the violence enacted upon these bodies.^(12,13)^ Furthermore, this distinction serves to delineate who is considered a subject of rights and who is not – whose ways of living can thrive under the colonial regime and whose cannot. For coloniality to succeed, it must rely on transparency^**^ – on the complete assimilation of the Other – which means that non-modern^***(14)^ forms of organization, whether pre-colonial or still resisting today, must be stripped of legitimacy and eliminated through an architecture of domination.^(12,15)^

We argue that the central idea of colonization is the proliferation of its own modes of life and organization at the expense of those that cannot be translated into its ontology. This is because it is only through the naturalized reproduction of coloniality that the structures of power sustaining the capitalist regime of cisheteromasculine whiteness can be established and maintained. For this to occur, multiple tools of domination are mobilized, such as the colonial demarcation of gender, which is only valid for universalized bodies, that is, white bodies. Thus, the concepts of “man” and “woman” become emptied of meaning when applied to colonized bodies, reducing these subjects to less than human, and therefore subject to colonial domination and violence.^(12,16)^

The coloniality of gender is, then, a material exercise of power – interconnected and expressed in various forms – that operates both through embodied subjectivity and at the institutional level, by defining who is included within the category of the human.^(12)^ Therefore, it is necessary to reflect on how this is produced and reproduced within the field of health – understood here as a contested space within the process of disciplining bodies, but also in the ongoing dehumanization of dissident existences. In this regard, Lugones^(12)^ highlights how coloniality circulates through all aspects of life through power. We propose that coloniality not only infiltrates life, it also constitutes it, since the ways life and institutions are organized are deeply shaped by the marks of modern colonial thought, with its categorical, dichotomous, and hierarchical logic.

Just as racial marking was fundamental for the colonial regime – and, consequently, for the capitalist regime as well – to prosper through structures of domination and material expropriation of territories such as Africa and South America, the dichotomous and binary distinction of gender was also essential for establishing power structures in ways that organized social life through the assignment of distinct gendered functions. This means that the coloniality of gender constituted a foundational marker in the rigidification of the differentiated social contributions attributed to men and women.^(12)^

However, the distinctions delineated by the coloniality of gender alone are insufficient to account for the complex ethical agenda of dehumanization inherent in the colonial process. This is because the hierarchization systems that structure social dynamics become more complex when we consider dissident existences within the sex–gender system, given that cisheteronormativity is also a defining feature of this political project. Cisheteronormativity is responsible for structuring biologizing fictions that give rise to tools such as compulsory heterosexuality and other mechanisms of cismasculine control over deviant bodies. This operates with the aim of ensuring that the reproduction of the species – in the service of capital structures – also entails the reproduction of their ontological assumptions, which are organized around a universal subject who is white male, as well as simultaneously cisgender and heterosexual. Thus, reproducing the species also becomes reproducing cisheterosexuality.^(12,17)^

In this sense, it can be stated that the coloniality of gender and cisheteronormativity are operative tools of the same system, distinguished by the ways in which they construct different mechanisms of material and symbolic privilege for hegemonic sexualities.

To help us think about how coloniality leaves its marks on the organization of life, we draw on the idea of transparency as a Western ontoepistemological foundation of modernity, instituted through the colonial process. This refers to the idea that all things must – and can – be explained rationally, universally, and scientifically. Thus, everything must be made translatable according to the criteria of intelligibility in Western modernity. Transparency becomes one of the lenses through which we can analyze the erasure of other ways of organizing the social and the cosmological, since, through this logic, all that cannot be translated into Western knowledge systems is dismissed as obscurantist and regressive.^(15)^

Whereas Glissant emphasizes the ethical and political significance of opacity and the limits of intelligibility in the recognition of experience, Gonzalez directs attention to the historically grounded and materially embedded ways in which racism and sexism operate in Latin American societies, demonstrating that care practices are never neutral; they are traversed by hierarchies that assign differential value to bodies, knowledge, and social contributions, rendering invisible those experiences that diverge from normative, hegemonic scripts of being, mothering, and caring.^(15,17)^

In this sense, we can start questioning the relationship between coloniality and the use of medical protocols in relation to the experiences of these women who chose to breastfeed. What are the effects of the use of protocols concerning the development of healthcare strategies for LGBTQIA+ individuals? In what ways might a form of care organized around the standardization of experiences contribute to the erasure of plural modes of living? What are the impacts of the protocol-centered model of care on the healthcare field? Finally, in what ways do protocols make evident the transparency assumptions of coloniality that sustain and structure healthcare practices?

In response to the universalization of Western transparency, Glissant^(15)^ proposes the idea of the right to opacity as a relational ethic, defending the notion that existence must be possible without the need for explanation or translation within the frameworks of coloniality. Opacity is thus the right to an irreducible complexity – intended to protect the singularity of subjects and communities.^(15,18)^

We ground our initial reflections on the impacts of colonial thought on the social organization and the construction of imaginaries surrounding breastfeeding, as well as on the formation of the medical field. However, we take the right to opacity as a philosophical horizon for imagining other ways of constituting care. It is from this perspective that we seek to reflect on the complexities of the use of lactation induction protocols based on the experiences of couples of cisgender women who have chosen double breastfeeding.

### Methodological articulations for our reflective exercise

This article is derived from a qualitative research study, whose general objective was to narrate and analyze how couples of cisgender women perceive and give meaning to the experience of double breastfeeding, with the analysis based on feminist authors from the Global South, in line with a process of constant positionality, reflecting the negotiations that occur in the fieldand an understanding of the complexity of the ethical commitment of social research with vulnerable participants.^(19)^ This study was submitted to and approved by the Ethics Committee of the *Faculdade de Saúde Pública, Universidade de São Paulo* under the CAAE: 6141523.8.0000.5421; # 6.598.184.

To contact participants and inform them about the research, a search was conducted on social media using hashtags such as #duplaamamentacao, #amamentacaocompartilhada, and #duplamaternidade – #doublebreastfeeding, #sharedbreastfeedinng, and #doublemotherhood, respectively. After identifying potential participants, initial contact was made via message on the platform itself. Additionally, key contacts, built throughout previous research on a related topic, were activated to help publicize the project. After recruiting the first participants, the snowball method was adopted to increase the sample.^(20)^

After the initial contact, the research was presented to participants, and it was possible to verify whether the couples met the inclusion criteria, which were that the women were currently or had previously undergone double breastfeeding, with one of the mothers having undergone lactation induction. For data production, semi-structured interviews were conducted – between June and October 2024 – via video calls lasting an average of 40 minutes. Their central objective was to understand the meanings and significance attributed by these women to their experiences, thus making this method suitable for data production. The Informed Consent Form was read and signed at the beginning of each interview, which was audio-recorded with the participants’ consent and later transcribed for analysis.

In qualitative research, interviews, whether open, semi-structured, or life histories – are powerful methods to access subjects’ discourse about their own narratives. This happens because the subjectivity of participants and researchers engage in dialogue during this encounter, enabling openness and exchange.^(21)^ At the same time, the scientific rigor of interviews is also ensured through tools such as descriptions of the meetings with participants, the description of the interview guide, and the ethical commitment of the researcher to acknowledge their positionality in all materials derived from the study.^(19)^

The semi-structured guide began with questions aimed at understanding who the participants were, such as name, age, and place of birth, and then covered three central topics: 1) the fertilization process and negotiations around it; 2) planning of shared breastfeeding and planning for it to happen; and 3) the practice of breastfeeding and the lived experience.

To begin the analysis, we propose a broad research question: What is the experience of following lactation induction protocols for cisgender women who opted for double breastfeeding? To answer this question, we used thematic content analysis according to the procedure proposed by Burnard.^(22,23)^ This consists of several stages, beginning with transcription and systematization of the interviews, including a period of immersion in the material produced and familiarization with the transcripts by the first author.

From there, open coding of the data was initiated, with an inductive and free approach to constructing themes so that in a later stage similar themes could be grouped, producing broader categories; the defined themes were then discussed and agreed upon with the second author. With the grouped and consensual data, we then proposed reflection and approximation of the material with the theoretical framework.

Based on an understanding of the dimension of the colonial wound in the constitution of ways of thinking and organizing life in the Global South, and understanding breastfeeding as life production, we mobilized as theoretical references authors who think about coloniality from different perspectives – such as Lugones,^(12)^ Quijano,^(24)^ and Glissant.^(15,18)^

### Reflexivity and positionality****

To the extent that the construction of research positions the researcher’s body in relation to their object within a power dynamic that is also rooted in the organizational modes proposed by coloniality, researching itself becomes a fundamental element to be investigated. Modern science, when it claims to be neutral – and relies on the fallacious duality between science and politics – exempts itself from the task of declaring which world project its interests serve, denying that knowledge is always situated and politically implicated.^(25,26)^ This is because the myth of scientific neutrality is also a tool in maintaining a successful colonial project; consequently, it assumes as true the figure of the universal being – man, white, cisgender, heterosexual – in sustaining the hegemonic power of coloniality.

We use modern scientific thought as an almost dichotomous example not to oppose or ignore the disputes in the field, but to build, through tension, another ethical implication. This is because being an LGBTQIA+ body engaged in research involves different intersections – here recognizing that the form and extent of violence will be shaped by other social markers. Therefore, the proposition is not one of ontological distinction but of lived experience in the body – through scenes of subversion, resistance, violence, and exclusion – thus it is important to bring identity also as an element of elaboration. Recognizing this is also recognizing that it is possible to establish another kind of relationship, negotiating with participants through different elements opening space for the possibility of other alliance politics.^(19,27)^

These elements are therefore fundamental when considering how the research object – which sometimes emerges from one’s own life experience – takes shape when it becomes research – since becoming a researcher is also constituted by constant negotiations between one’s own reality and the research. Thus, engaging with scientific work in a way committed to the pursuit of social justice necessarily involves reflecting on the structures on which the ontoepistemological assumptions that guide and particularize the research process are founded.

These elements are fundamental to understanding how this research is negotiated in the field, because as a researcher, my ethical and epistemic commitments are influenced by my subjectivity and consequently by my political alliances. Being a woman who also forms relationships with women and who, from an early age, had her body marked by homophobic reactions to dissident gender performances plays an important role in constructing a political dimension of my own existence. Recognizing this is acknowledging that when looking at other scenes of life production and/or violence that are crossed by dissidence of the sex/gender system, affects in my body will be, and have been, mobilized, producing new meanings in the observed reality.

It is necessary to recognize and to position my body as an element that carries with it a history because it is from it that proximities and distances are constructed in relation to the women participating in my research, facilitating or producing tensions in the field. Thus, being attentive and vigilant to these affects and, understanding the importance of their records in a field diary became fundamental in the research process so that it would be possible to dialogue with an understanding of the power relations built from the researcher-participant dynamic.

The data from this study will be made available by the authors upon reasonable request. This is because part of the content shared by participants is confidential in nature, and full disclosure of the data would, for some of them, inevitably compromise their anonymity.

## Results and discussion: between the protocol and opacity

Seven women participated in the research, comprising three couples and one non-gestational mother whose former partner declined to participate; one interview was conducted with the couple together, and the others were conducted individually with the participants. All participants self-identified as cisgender and white; two participants reported being lesbians, one reported never having had relationships with men, one self-identified as pansexual, and the others did not disclose their sexual orientation. Regarding the pregnancy during which they practiced shared breastfeeding, four participants were non-gestational mothers, and three were gestational mothers. The participants’ names were changed to preserve their identities; Table 1 presents the sociodemographic data of the participants. All names were changed to respect the privacy of the participants.

**Table 1:** Sociodemographic data of the participants

	Julia	Gabriela	Lucia	Suzana	Fabiana	Marcia	Bruna
Age	35	34	52	39	29	30	37
State of residence	São Paulo		São Paulo		Marília		Niterói
Profession	Physiotherapist		Musician	Actress and translator	Kitchen assistant		Nurse
Declared monthly income	5 minimum wages		4 minimum wages		3.5 minimum wages		7 minimum wages
Race/ethnicity	White	White	White	White	Not disclosed	Not disclosed	Not disclosed

For the development of the discussion, two categories of analysis were mobilized and systematized in table 2, which includes the name of each category, a brief description, and a typical statement from a participant.

**Table 2:** Analysis categories constructed from the research objective: to explore the complexities of the use of lactation protocols for cisgender women couples who chose shared breastfeeding

Category names	Brief description	Typical example
Sources of information about the protocol	Adresses the disponibility, access and popularization of information about the professionals involved, the protocols and the double breastfeeding	“And… information, uh… on the internet, with people we met along the way who breastfed, I’m also in a support group, it’s along those lines.” [Lucia, non-gestational mother]
The routine and the protocol	Adresses the perception and the lived experience of these women about following the protocols while living their routines.	“I wanted to do it as healthily as possible and everything, so I really committed myself. I was pumping every two hours, even during the night. I’d wake up every two hours as if there was a baby nursing there. I’d pump for 30 minutes on each side until I bought a double pump that did both at the same time to save time. I worked in front of the computer with the pump on because I really needed the stimulation during the day [...]” [Bruna, non-gestational mother]

We mobilized the categories according to what emerged from the interviews, to explore some aspects of the relationship between the possibility of shared breastfeeding through IL and the use of a protocol designed by and for cisheterosexuality. We understand that the availability of information about lactation induction protocols emerges as a central issue, since adherence to this practice is only possible when individuals are aware that it is an option.

In this sense, the internet was pointed out as the main source of information and/or the first contact these women had with the possibility of breastfeeding together:

“Later, I read on the internet itself, I am not exactly sure where now, but I read that it was possible to do induction with medication, though I never knew what medication it was. So much so that during the process of these five years trying to get pregnant and everything, I always told her [my partner]: ‘hey, it would be cool if you breastfed too because you have an extra connection with the baby…’” [Bruna, non-gestational mother]

Social media, specifically, also emerged as a central tool not only for access to information but also for creating networks of experience exchanges through real-time follow-up of other people on their journey with lactation induction or even breastfeeding:

“During the fertilization process, we started following Julia Apolinário [digital motherhood influencer], who went through fertilization [...] at the same clinic we used. We actually found the clinic through her – like, a random video popped up, I watched her talking about the fertilization process, and then we started following her. Then, if I am not mistaken, she posted a video about shared breastfeeding. That’s when I started following Roberto Bete [trans man, digital influencer], and he was married to Erika [trans woman, digital influencer], and Erika breastfed their child. So we started researching about it, and we kept following… pages that [...] talked about shared breastfeeding, and we started looking into it and then decided to try.” [Marcia, non-gestational mother]

Further, we delve deeper into public figures who appear as important references in this process for the research interlocutors. However, at this moment, we discuss what we found lacking in the responses. Now, if on the one hand the internet and digital influencers of parenting were identified as the main source of information for these women, we must ask ourselves where the health professionals are and why they do not appear as sources of information or are not even mentioned when we ask participants about the sources they turned to. If what emerges from the interviews is fundamental data, what does not appear also ends up shedding light on a certain disinterest of the field in looking at certain bodies and, consequently, their care strategies. The fact is that sexual and gender dissidences intertwine with medical history, especially the history of psychiatry, through violence – such as the pathologization process and conversion therapies – and not through care, which will impact the ways medicine produces or fails to produce health for this specific population.^(28)^

We can follow different elaborations to think about this absence, but here we are interested in looking at two issues: 1) the way that the lack of health professionals trained and engaged in LGBTQIA+ breastfeeding leads these women to construct new tools for disseminating and accessing the necessary information to make the induction process possible, and 2) how this absence reflects the colonial assumptions that also underpin the fields of Health and Medicine. If modes of social organization are permeated by the need for absolute transparency, which has modern rationality as its ontological principle, then care practices established from other rationalities will not be apprehended by the scientific field and, therefore, by the field of medicine and health. Here, what we mean is that the desire for dual breastfeeding cannot be understood by the canon of the health field, since affection – as well as strategies of resistance to the way of life under the capitalist regime – are not founding principles of the field and, therefore, escape its structure.^(13,15)^ This, because coloniality operates in favor of capital and the maintenance of its power, which means also reproducing its ways of organizing the social based on cisheterosexuality.^(15,18)^

Thus, acting in favor of the hegemonic, it is a fact that Health will concern itself somewhat less with care practices that are based on other ways of organizing the social, through collective care strategies that challenge rigid structures of domination, a situation intensified by cisheteronormative structures, which will define that the reproduction of life must be the reproduction of heterosexuality and its assumptions.^(27)^ This is because for the colonial ontology to thrive, non-modern modes of social organization must be rendered invisible.^(12,24)^

In any case, just as for Lugones,^(12)^ it is important to look at resistance as a starting point for the production of a creatively oppositional liberation, thus understanding the ways in which communities organize themselves in rejecting the meanings and modes of social organization designed by power structures. We are interested in looking at this production of life that resists cisheteronormative organizations. As an example of this, the interviews also bring examples of visibility that insist on escaping silencing logics:

“Marcela Tiboni! [...] A friend of mine who is a doula, who wasn’t our doula, but a friend of mine way back [...] she sent me the invitation to the launch of *Mama* [...] We went, my partner and I. We went to the book launch, bought the book, met the girls, sat in a discussion circle with Kelly [lactation consultant] and Natália, and then I do not know what we talked about, we talked and talked… [...]. Yeah… and that’s how Lucia started thinking about breastfeeding.” [Suzana, pregnant mother]

The book *Mama: um relato de maternidade homoafetiva*^(29)^– which narrates the author’s induced lactation (IL) process and her partner’s pregnancy – seems to be a landmark in the popularization of the practice of shared breastfeeding. This is because the author, Marcela Tiboni, along with her lactation inducer, Kelly Camargo, frequently appeared as important figures in the learning journeys of the research interlocutors regarding their decisions about shared breastfeeding. Thus, the internet and groups of women who go through similar experiences emerge as an important network for information exchange. Although belonging to a group – here, of women in relationships with women – is not in itself a resolution to the issues of erasure and exclusion organized by the State as a power structure and by the health field, there lies a potential for proposing dialogue among people that can produce care networks established through encounters with others.

Dialoguing with our second category, when we look at the practice of shared breastfeeding through the IL process, we see a particular complexity. This is because protocols contribute to what Berg^(30)^ calls *the illusion of a single answer*, which corroborates the idea that rigidly following the protocol guarantees the desired outcome, as strongly stated of one of the interlocutors:

“I remember a consultation we had, I think it was in June, when we had this consultation with the lactation consultant. And I told her, in front of Lucia [the partner], I said, “I’m afraid she’ll get frustrated… because she’s not stimulating, she’s not expressing milk, she’s not doing what’s indicated, and when the time comes, there are all the issues, difficulties…” especially in the beginning until breastfeeding is established for the one who gestated, and then establishing the bond with the one who didn’t gestate, there’s a whole adaptation process, I said, “I think Lucia won’t be able to handle this emotional and psychological process, and I think it could be very bad for her.” I called it, didn’t I? Because that’s exactly what happened.” [Suzana, pregnant mother]

This discourse is also rooted in the narrative constructed around breastfeeding, based on individual blame placed on the one who does not meet the expectations built around her own experience – all in the name of a supposed healthcare model that centralizes its impact on the child’s health, without even considering the reality of these women. This brings us to another important point regarding the use of protocols: the frustration generated when following the protocol is not possible due to different aspects of these women’s lives given that carrying out the protocol demands a certain amount of time and dedication, which often does not align with how these women’s lives are organized, leading to discomfort and frustration,^(30)^ as we can discern from the account provided by Lucia, Suzana’s partner:

“So there was this whole procedure, this whole ritual, this whole process that I couldn’t follow exactly, so sometimes it was uncomfortable, having to do so many pumpings, so much... right, the process for me was harder. I knew it was for a positive result, so I did what I could. I tried to make it work, but sometimes I got tired of putting that machine on my chest to keep pumping; it’s uncomfortable, physically speaking.” [Lucia, non-gestational mother]

It could be argued that since this is a woman’s choice, she should then be prepared for the procedure. However, it is necessary to understand other dimensions of what a protocol means, as it is the result of a mode of health care production that seeks to standardize experiences without considering social, cultural, and subjective dimensions and, simultaneously, without addressing the macrostructures of Capital. The protocol is indeed an important tool in the induction process, as it is through it that the possibilities for shared breastfeeding are opened. However, its use also brings tension to the biomedical standardization that tends to fit experiences into a mold by focusing only on universal subjects.

Glissant^(18)^ argues that the Western way of universalizing transparency as a fundamental condition for existence is also a form of epistemological domination, and this ends up impacting how care is produced through public policies. Thus, it is necessary to know everything about “the other” so that it becomes possible to categorize them and, therefore, assimilate them. This need imposed by coloniality to translate the ways of life of colonized subjects into explanatory models that serve dominant logics is also a way of guiding knowledge production, thereby shaping the institutions derived from it. This means that transparency here acts not only as a tool for the subjugation of the colonized other but also as a constitutive element of the Western way of thinking and producing knowledge.

Let us return, then, to the protocols; now, if transparency is fundamental to the assimilation of subjects, it is also fundamental to the production of health strategies. Here, let us consider two fundamental tools of the colonial mode of organizing life: 1) the coloniality of being, responsible for establishing the white European cisheterosexual man as the reference; and 2) the universalization of transparency as a mode of organization – along with the denial of that which cannot be translated or encompassed by its own logic. These two tools then give us clues about how protocols are designed: based on a universal subject who is not a reflection of their time and territory, and on a crystal-clear way of thinking that leaves no room for the creative production of care in action.^(18,24)^

Thus, lactation induction protocols occupy a paradoxical position within healthcare practices directed at couples of cisgender women: while they constitute a fundamental tool for enabling the experience of shared breastfeeding – particularly in biomedical contexts historically organized around heterocentered motherhood – they also operate through a logic of standardization that tends to silence the singularities of lived experiences. By relying on universalizing parameters, these protocols often disregard the bodily, affective, social, and symbolic differences that shape care processes, thereby producing a tension between access and recognition. Thus, while protocols expand possibilities by legitimizing dissident practices within institutional healthcare settings, they simultaneously reveal their limitations by failing to fully account for the plural ways of being, mothering, and breastfeeding. This contradiction highlights the need to conceptualize care beyond exclusively protocol-based structures, incorporating approaches that acknowledge the contingency, relationality, and opacity inherent to experiences that escape normative models.

This is also not an argument that ignores the attempts at dialogue within protocols, such as the one published by the ABM,^(1)^ regarding individual needs. However, it is necessary to consider how much space is left for practices and knowledge that arise from the territories, as well as for the lived realities of these women, when unique and singular experiences such as the lactation induction process, are turned into protocols.

Therefore, drawing on Lugones,^(12)^ it is necessary to consider the extent to which there is space for a creative-oppositional liberation built through resistance to the coloniality of gender.

In this sense, a subjective gaze toward the production of life in action becomes fundamental, since it is through the lived experience within the territory that one can perceive the micropolitics that resist dominant structures and open up space for creativity. As an example of this, a lactation induction professional appears in several interviews as a positive deviation in the interlocutors’ experiences with healthcare professionals, by proposing adaptations to the protocol and creating room for dialogue centered on the unique experience of that specific body.

“And then we did a home insemination, and Levi came right away. So, before the attempt, I was already taking medication, the one that stimulates the stomach. [...] Then we found out about the pregnancy on July 19th, and on July 23rd, the first drop of milk came out, and from there, I just kept stimulating. [...] Domperidone, which is the medication used, I already knew it had this effect of increasing your prolactin, but I wasn’t taking it with that intention, then from the moment I saw the first drop, I thought: this is it, this is the chance. This is the beginning, right?! I’ll extend this, so I started manually stimulating on my own, and then later I had help from Kelly and everything else… [...] She didn’t follow the protocol with birth control medication, nothing like that, because I was already producing, so from then on I increased [...]..” [Bruna, non-gestational mother]

As another non-dialogical aspect of the protocol experience, the idea emerged from the interviews themselves that individuals who opt for dual breastfeeding must have bodies that are ready and willing to go through IL, strictly meeting the requirements of the protocol:

Therefore, claiming what is already granted to cisheterosexuality – such as the right to breastfeed – is not enough for us to develop care strategies centered on the differences produced by dissidence. For that, it is necessary to open up space for political creativity so that opacity becomes the foundation of our relational ethics, producing spaces where the existence of subjects is more than a constant process of assimilation.^(13,15)^

## Final considerations

It is important to note that this study represents only a partial perspective on the realities of the diverse women who experience double motherhood and double breastfeeding. By working with a small sample, we do not intend to produce universal knowledge – not only because this has historically been the role of colonial epistemology, but also because we radically defend the preservation of diversity. Thus, acknowledging the study’s limitations is fundamental, as it allows us to reflect not only on care grounded in protocol-based structures, but also on the assumptions that organize the field of scientific knowledge production.

In this regard, it is noteworthy that most participating women self-identified as White, while three participants did not provide racial self-identification. This question was part of the initial block of interview questions, and participants were asked more than once and in different ways about this information; however, when they expressed discomfort with the question, we did not consider it ethically appropriate to insist on racial self-identification. The lack of greater racial diversity among the interviewed women therefore produces a particular effect on the data, as our analysis ultimately centers on the specific realities of White women – or women without declared race – thus generating a new gap: how do Black, Indigenous, or Asian women experience double breastfeeding, and what distinct forms of intersection emerge from non-White racialization?

It should also be noted that, during the process of active recruitment and sample sensitization, there was a concrete effort to ensure greater racial diversity. However, we encountered a specific challenge: when contacted, two different couples of Black women reported being unable to participate in the study due to the lack of financial compensation for the time required for the interview, as well as difficulties in organizing childcare, paid work, and dedicating a specific time to the meeting. This led us to materially confront the effects of colonial structures that render invisible the histories that coloniality does not wish to amplify. Consequently, we are left with the task of creatively cultivating new possibilities for engaging with and constructing the field, allowing us to imagine alternative pathways, given that even the assumptions underlying research ethics principles are deeply connected to a colonial ontology of erasing dissident experiences.

Reflecting on the complexities of using protocols through the lens of women’s experiences with double breastfeeding also means reflecting on what underpins our practices of health production. To do so, we draw from two categories that emerged from the data. The first relates to the sources of information used by the interlocutors, highlighting key actors in this process, such as the information networks built through the internet, and the absence of health professionals as reference figures in this process. The second category discusses these women’s experiences with the induction protocol, pointing to the difficulties in reconciling their routines with the activities related to the protocol, and the importance of professionals who act as positive deviants from the hegemonic field of care production.

In this way, it becomes possible to think that healthcare is founded upon a colonial logic – therefore binary – rooted in the dichotomy between humans and nature, designed by and for the colonial subject alone. Thus, care strategies and ways of organizing life that escape this logic and are not based on the structures it upholds – church, family, marriage – cannot be captured or understood by Western organizational frameworks.

In this specific context, we can bring Gonzalez’s perspective to help us problematize the tension between accessibility and standardization. While such protocols enable shared breastfeeding among couples of cisgender women, they simultaneously reproduce inequalities by imposing universalized procedures that fail to account for the singularity of experiences, particularly those shaped by racialized and classed positions. In this sense, Gonzalez complements Glissant and Lugones by foregrounding the concrete, historical, and material dimensions of exclusion, showing how systemic hierarchies determine which bodies are authorized to participate in care, which practices are recognized as legitimate, and which experiences are silenced.

In this sense, we can return to the questions presented in the introduction to articulate the relationship between coloniality and the use of medical protocols in relation to the experiences of these women who chose to breastfeed together. Although protocols emerge as an important instrument for expanding care perspectives – providing a step-by-step pathway toward a desired clinical outcome – their application generates specific effects within healthcare practices. This occurs because their use reinforces the notion that health-related issues are amenable to resolution or management through a single, standardized directive; in doing so, it undermines opportunities for dialogue and co-construction between healthcare professionals and users/patients and constrains the creative possibilities that might otherwise arise from the singularities inherent to each case. Moreover, the hypervaluation of protocols as a central tool reflects a broader tendency toward bureaucratization and regulatory intensification that increasingly structures healthcare practices. It becomes evident, therefore, that even the principles underpinning the development and deployment of medical protocols are grounded in ideals of transparency and the homogenization of experiences, ultimately oriented toward the production of universalizing healthcare strategies.^(30)^

Therefore, we can understand nonassimilation into colonial logic as approaching what Lugones^(12)^ calls resistance, which for her is the tension between subjectification (the formation of the subject) and active subjectivity (a minimal agency), enabling the relationship between oppression and resistance to become an active one, and therefore, productive of another lived reality. Clearly, there is no intention here to defend assimilation into capital as a pathway toward building a politics that accommodates our marginal existences. What we advocate for, in fact, is the dismantling of colonial modes in healthcare practices, offering as a philosophical tool the idea of the right to opacity proposed by Glissant.^(15,18)^

Thus, we propose to the field of health a reflection on its own onto-epistemological assumptions, understanding that its knowledge system does not – and will not – on its own, grasp the realities of life in the territory, especially where social organization occurs outside modernity, where care is collective, dissidences circulate, and other modes of articulation underpin resistance to capital. Moreover, we propose that the right to opacity become one of the horizons for the construction of practices within the field of health, not only in clinical practice, but also in scientific production. It is not necessary to know everything about the other or about their ways of organizing life in order to make an alliance of care possible, as opposed to protocol-driven framing.

## Footnotes

* This refers to the development of medical technologies and equipment, and the expansion of the pharmaceutical industry, creating a scenario of high consumption of medical services.

** In this paper, the term “transparency” is used solely in the sense proposed by Glissant.

*** Here, the definition was borrowed from Aparicio and Blaser.

**** The reflections on the processes of reflexivity and positionality presented in the text refer to the first author’s personal process.
